# An Unusual Cause of Subacute Headache in a Patient Undergoing Chemotherapy for Advanced Testicular Nonseminomatous Germ Cell Tumour

**DOI:** 10.1155/2016/4317108

**Published:** 2016-04-21

**Authors:** Helen M. Clarke, Ankit R. Rao, Emilio Porfiri

**Affiliations:** The Cancer Centre, The Queen Elizabeth Hospital, Edgbaston, Birmingham B15 2TH, UK

## Abstract

Testicular (germ cell) cancer is a model of a chemocurable malignancy and tends to have a favourable prognosis even in advanced stages due to exquisite sensitivity to platinum-based chemotherapy. However, both acute and longer-term toxicities of multiagent chemotherapy remain significant as causes of morbidity, very occasionally mortality, and impaired quality-of-life. Here, we report a case of acute cerebral venous sinus thrombosis occurring within 10 days of chemotherapy initiation in a young patient without comorbidities, whose only predisposing factors were malignancy, chemotherapy, and perhaps mild dehydration. The clinical presentation was also unusual with headache of moderate severity only without focal or global neurologic deficits. We suspect that cisplatin may have had direct vasculotoxic effects. The patient recovered fully after short-duration anticoagulation but oncologists must remain aware of unusual and unpredictable complications of cytotoxic treatment.

## 1. Introduction

Testicular cancer is the commonest solid malignancy in young men and since the development and application of cisplatin-based cytotoxic chemotherapy [1] in the 1970s the outlook, particularly for patients with metastatic disease at presentation, has been transformed with cure rates in excess of 85%. In view of the excellent outcomes in most patients except those with poor-risk features such as liver, central nervous system, or osseous metastases [2], a current focus has been on reducing the chronic, and to a lesser extent acute, toxicities of chemotherapy and also on survivorship issues. Improvements in supportive care such as the introduction of 5HT-3 (e.g., ondansetron [3]) and NK-1 (e.g., aprepitant [4]) antagonists have dramatically reduced the severity of emesis, and the use of granulocyte colony-stimulating-factors has reduced rates of neutropaenic infection [5]. However, longer-term side effects such as neuropathy, vascular damage including Reynaud's phenomenon, subfertility, secondary myelodysplasia, and pulmonary fibrosis remain significant problems. In the acute setting, cisplatin is classically associated with acute arterial thrombosis affecting the myocardial, ophthalmic, and cerebral vascular supply [6]. In this case, we describe an unusual site of extensive venous thrombosis occurring early after chemotherapy for advanced testicular cancer with a favourable outcome.

## 2. Case Presentation

**Figure 1:**
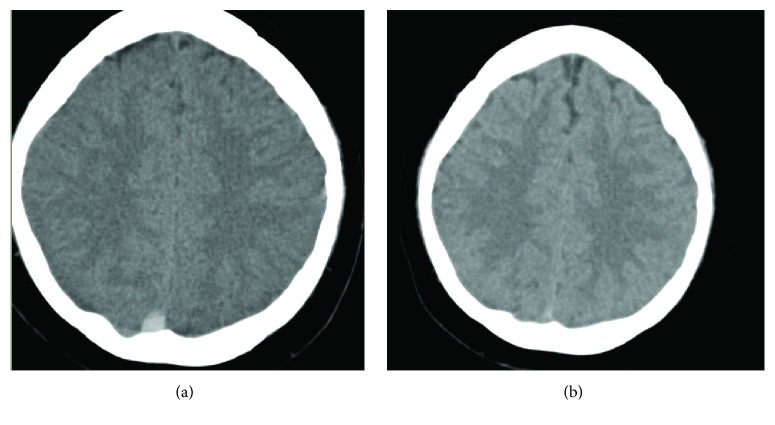
Unenhanced CT scan of the head (a) showing acute hyperdense thrombus close to the torcula and (b) complete resolution 4 months later following therapeutic anticoagulation.

A 22-year-old man, of South Asian ethnicity, with a known diagnosis of Stage IIIA (pT1, cN2, and cM1a) nonseminomatous germ cell tumour was undergoing multiagent cytotoxic chemotherapy with curative intent. He had no significant medical comorbidity but was a cigarette smoker and moderately overweight. He had undergone right inguinal orchidectomy which revealed predominant (99%) embryonal carcinoma (immunohistochemistry positive for placental alkaline phosphatase, cytokeratins, and CD30) with a minor (1%) malignant teratoma intermediate component. Staging CT scan showed lymphadenopathy in the retroperitoneum, pelvis (right external iliac), superior mediastinum, and left axilla. Four days after the first dose of intramuscular bleomycin as part of the bleomycin, cisplatin, and etoposide (BEP) chemotherapy regimen, he complained of recent onset headache over a 7–10-day period. He described it as a generalised, moderately severe constant “pressure” headache that was worse on waking in the morning and exacerbated by coughing and straining. There were no other neurologic symptoms. He had also vomited on two occasions in the first 5 days following cisplatin and had several episodes of loose stools. On examination, there was no focal neurologic deficit and he was alert and orientated. Laboratory investigations showed mild hyponatraemia (133 mM), raised serum urea (7.8 mM), and profound leucopaenia (0.7 × 10^9^/L) and neutropaenia (0.0 × 10^9^/L), and moderate thrombocytopaenia (53 × 10^9^/L) in keeping with recent chemotherapy administration. On the second day of admission he had a brief and self-terminating episode of loss-of-consciousness. Unenhanced CT scan of the head showed hyperdensity in the distal aspect of the superior sagittal sinus close to the torcula (Figure 1(a)) and a subsequent CT intracranial venogram confirmed acute sagittal venous sinus thrombosis close to the torcula with complete occlusion and partial nonacute occlusion of the right transverse and sigmoid sinuses. There was no evidence of cerebral metastases. He was treated with low-molecular-weight heparin (enoxaparin 1.5 mg/kg once daily) administered subcutaneously and there was a rapid improvement in the headache over a week. Following three months of therapeutic anticoagulation without any haemorrhagic complications, repeat imaging confirmed recanalisation of the cerebral dural venous sinuses with disappearance of the hyperdense thrombus (Figure 1(b)) and anticoagulation was discontinued. Currently, the patient is in complete remission from his metastatic testicular cancer with regression of all sites of lymphadenopathy having completed all 3 scheduled cycles of BEP chemotherapy.

## 3. Discussion

Cerebral venous sinus thrombosis (CVST) is an uncommon condition that typically affects young females with obvious risk factors such as pregnancy or oral contraceptive pill use or patients with coexistent local inflammatory pathology or inherited thrombophilias. It is a rare cause of cerebrovascular disease and is probably underrecognised [7, 8]. In our patient, the description of the headache was entirely consistent with raised intracranial pressure as the cause and the subacute chronology was also typical of CVST. However, the lack of focal (e.g., hemisensory loss and hemiparesis) or generalised (e.g., decreased conscious level) neurologic deficits was unusual as between one-third and three-quarters of patients do present with these [9] and perhaps suggests that the diagnosis was made early in the disease process' evolution. The predisposing factors in this case include the presence of active (albeit highly chemosensitive) malignancy, the direct prothrombotic effect of chemotherapy (in particular cisplatin which can be associated with vascular damage), and a degree of dehydration due to mild chemotherapy-induced diarrhoea and nausea as evidenced by the raised urea levels and hyponatraemia. His body mass index of 31 kg/m^2^ and cigarette smoking may have contributed. Interestingly, extensive thrombosis developed in a highly unusual anatomical site despite moderate thrombocytopaenia after chemotherapy. In the presence of risk factors, the patient was not screened for inherited thrombophilic tendencies (e.g., Factor V Leiden and antithrombin III deficiency) although this should be considered prior to anticoagulation in unprovoked CVST. He had no family history of recurrent venous thromboembolism or miscarriage in female relatives. CVST has been previously described as occurring in various malignancies including cervical cancer [10], colorectal cancer [11], and Ewing's sarcoma [12]. In keeping with the features of our case, CVST occurring early after the commencement of BEP chemotherapy has been described [13], perhaps mainly attributable to the direct vascular endothelial toxicity of cisplatin.

Although venous thromboembolism (VTE) is common in patients with malignant disease, particularly those undergoing chemotherapy, it typically affects the lower-limb deep veins and/or pulmonary arterial tree leading to well-recognised and appreciated clinical syndromes. It remains important for the practising oncologist to be vigilant for unusual sites of venous thrombosis and to investigate and treat these in an expedient manner. We note that primary prophylaxis against VTE in cancer patients, whilst a reasonable consideration, has not been proven to improve overall survival in the setting of newly diagnosed lung cancer with an increase in bleeding events [14].

In terms of investigation, in this case we proceeded directly to cerebral imaging on the basis of the symptoms and clinical scenario, although measurement of plasma D-dimer/fibrin degradation product levels may have a role particularly in excluding CVST in low-moderate risk patients [15]. This case also illustrates that a comprehensive evaluation of possible causes of headache in these patients is essential, especially in patients without any personal history of chronic headache (e.g., tension headache, migraine, and cluster headache).

Our patient had a favourable outcome with the minimal duration of 3 months of anticoagulation with radiological evidence of thrombus resolution and disappearance of symptoms. However, there remains uncertainty as to the optimal duration of anticoagulant treatment in this rare disease; international guidelines suggest 3–12 months of therapy individualised for each patient [16]. The role of direct-acting, fixed-dose oral anticoagulants such as rivaroxaban in this setting remains to be proven and is under investigation.

In conclusion, although the outlook for patients with advanced testicular cancer is highly favourable, chemotherapy remains potentially hazardous as illustrated in this case and a high index of suspicion is required to assess for and manage complications to allow safe delivery of treatment.
